# The Antihypertensive Effect of Quercetin in Young Spontaneously Hypertensive Rats; Role of Arachidonic Acid Metabolism

**DOI:** 10.3390/ijms21186554

**Published:** 2020-09-08

**Authors:** Fawzy Elbarbry, Khaled Abdelkawy, Nicholas Moshirian, Ahmed M. Abdel-Megied

**Affiliations:** 1 School of Pharmacy, Pacific University, Hillsboro, OR 97123, USA; mosh6645@pacificu.edu (N.M.); dr_ahmed80@pacificu.edu (A.M.A.-M.); 2 College of Pharmacy, Kafrelsheikh University, Kafrelsheikh City, Egypt; khaled@pacificu.edu; 3 Pharmaceutical Analytical Chemistry Department, Faculty of Pharmacy Kafrelsheikh University, Kafrelsheikh City, Egypt

**Keywords:** quercetin, spontaneously hypertensive rats, arachidonic acid, metabolism, soluble epoxide hydrolase, hypertension, 20-HETE, EETs

## Abstract

Hypertension affects almost 50% of the adult American population. Metabolites of arachidonic acid (AA) in the kidney play an important role in blood pressure regulation. The present study investigates the blood pressure-lowering potential of quercetin (QR), a naturally occurring polyphenol, and examines its correlation to the modulation of AA metabolism. Spontaneously hypertensive rats (SHR) were randomly divided into four groups. Treatment groups were administered QR in drinking water at concentrations of 10, 30, and 60 mg/L. Blood pressure was monitored at seven-day intervals. After a total of seven weeks of treatment, rats were killed and kidney tissues were collected to examine the activity of the two major enzymes involved in AA metabolism in the kidney, namely cytochrome P450 (CYP)4A and soluble epoxide hydrolase (sEH). Medium- and high-dose QR resisted the rise in blood pressure observed in the untreated SHR and significantly inhibited the activity of the CYP4A enzyme in renal cortical microsomes. The activity of the sEH enzyme in renal cortical cytosols was significantly inhibited only by the high QR dose. Our data not only demonstrate the antihypertensive effect of QR, but also provide a novel mechanism for its underlying cardioprotective properties.

## 1. Background

Almost half of the adult population in the United States have high blood pressure, defined as a systolic blood pressure (SBP) ≥ 130 mm Hg or a diastolic blood pressure (DBP) ≥ 80 mm Hg, or as taking medication for hypertension [1]. Due to several socio-economic and pathological reasons, only 24% of patients with hypertension have their blood pressure under control [1].

**Figure 1:**
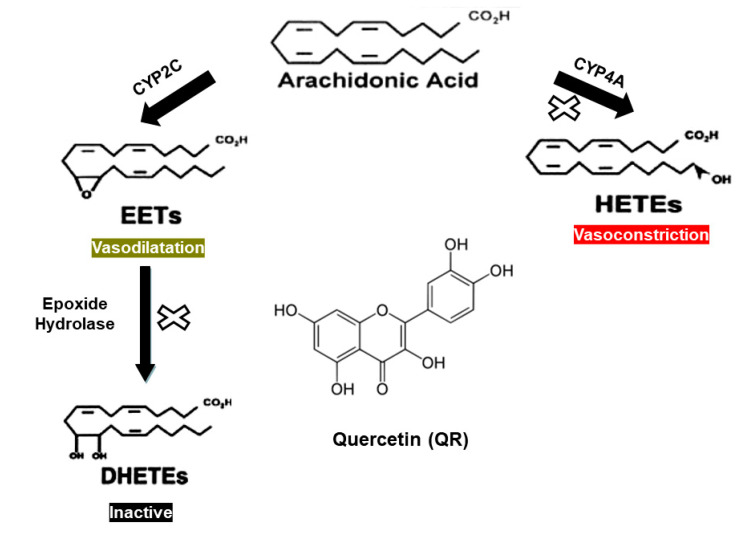
Pathways for cytochrome P450 (CYP)-mediated metabolism of arachidonic acid in the kidney. The present study examines pathways marked with the sign “x” as a potential mechanism for the blood pressure-lowering effect of QR.

Regulation of blood pressure is maintained through several integrated cardiac, vascular, neuronal, and hormonal factors. Evidence from numerous studies indicates that cytochrome P450 (CYP)-mediated metabolism of arachidonic acid (AA) in the kidney generates vasoactive metabolites that play a key role in the regulation of vascular tone and blood pressure [2,3,4,5]. The AA metabolites that have been extensively studied and demonstrated a significant role in blood pressure regulation are hydroxyeicosatetraenoic acids (HETEs; particularly 19- and 20-HETE) and epoxyeicosatetraenoic acids (EETs, Figure 1) [6].

Several studies have demonstrated the impact of 20-HETE in blood pressure regulation due to its potent vasoconstriction effect [5]. Although CYP4A11 and CYP4F2 are the major CYP enzymes responsible for the production of 20-HETE in the human kidney, CYP4A1 was found to exhibit the highest catalytic activity for the formation of 20-HETE in rats, followed by CYP4A2 and then CYP4A3 [7]. Blocking or reducing the rate of 20-HETE formation through using competitive inhibitors or antisense oligonucleotide against CYP4A1 has shown a blood pressure-lowering effect in animal models of hypertension [6,8,9]. Numerous studies have provided ample evidence regarding the implication of 20-HETE in the pathogenesis of high blood pressure in the spontaneously hypertensive rat (SHR) [10,11].

Epoxyeicosatetraenoic acids (EETs), on the other hand, are potent vasodilators mostly due to the activation of k^+^ channels and modulation of the activity of angiotensin II [12]. Reduced bioavailability of EETs has been found to predispose rats to blood pressure elevation [6]. Similarly, several new EET analogs have been developed and demonstrated positive effects in improving cardiovascular function and blood pressure control [13]. Although several CYP enzymes are involved in the formation of EETs from AA, the CYP2C family is responsible for more than 50% of the hepatic and extrahepatic epoxygenase activity in both humans and rats [14,15]. The biological effect of EETs is significantly limited by their rapid conversion to their corresponding and less potent diols (DHETEs), through the action of the enzyme soluble epoxide hydrolase (sEH) [15] (Figure 1). Chronic inhibition of sEH increases plasma EETs and exerts antihypertensive and cardioprotective properties in several animal models of hypertension, including SHR [16,17].

Our laboratory has been interested in exploring naturally occurring phytochemicals that have the potential to modulate AA metabolism (20-HETE-formation or sEH activity) and provide antihypertensive and cardioprotective effects. Recently, we investigated the effect of isothiocyanates and thymoquinone on drug-metabolizing enzymes and blood pressure [6,18,19]. In this study, we applied a similar approach to the natural phytochemical quercetin (QR), which is one of the most abundant flavonoids with a wide variety of therapeutic benefits including cardioprotection and anti-inflammatory effects [20]. The aim of the present study was to investigate the antihypertensive effect of QR in male spontaneously hypertensive rats following sub-chronic oral administration and to evaluate the impact of QR administration on the metabolism of arachidonic acid in the kidney, with particular focus on sEH and CYP4A.

## 2. Results

**Figure 2:**
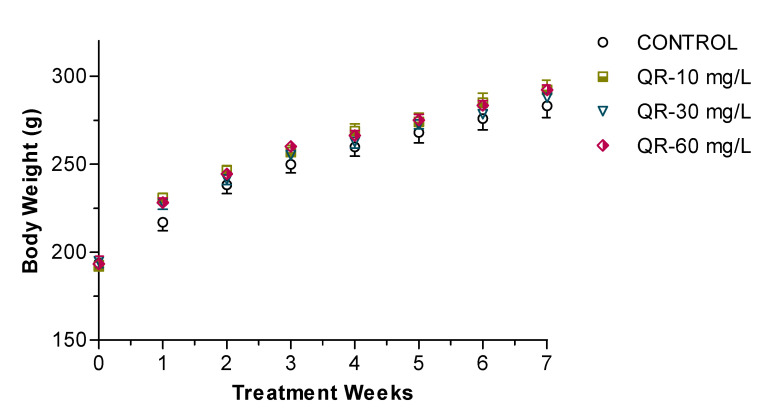
Average weekly body weight of control and QR-treated male spontaneously hypertensive rats. Data are presented as mean ± SEM, with each data point representing an *n* = 8.

Oral administration of QR for seven weeks had no obvious adverse effect on rats. The age-dependent increase in body weight was not significantly different in the QR-treated rats compared to control rats (Figure 2). Average daily water intake was assessed for each cage and determined as 18 ± 1.0 mL/100 g of body weight (*p* > 0.05).

### 2.1. Effect of Quercetin Treatment on Blood Pressure

**Figure 3:**
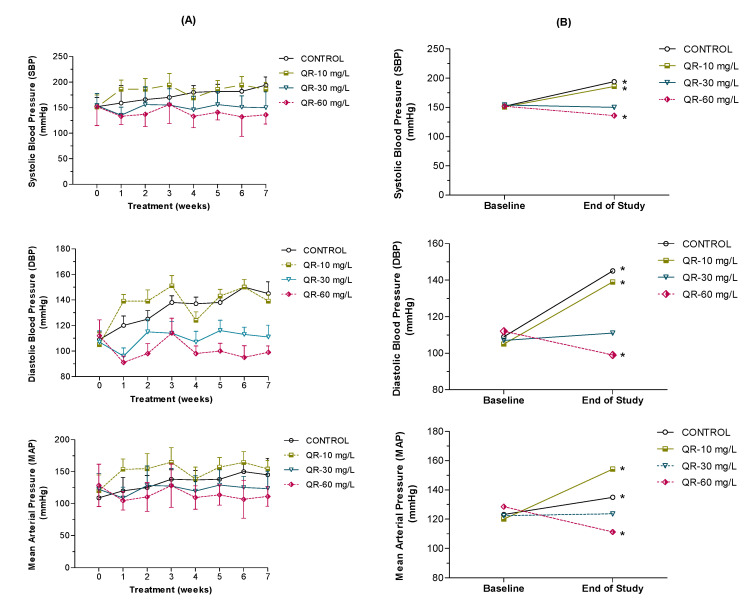
Average weekly systolic, diastolic, and mean arterial blood pressure of control and QR-treated male spontaneously hypertensive rats (SHR) (**A**). Changes in blood pressure in both control and QR-treated SHR rats during the 7-week study period (**B**). Data are presented as mean ± SEM (*n* = 8). * indicates significant difference, lower or higher, from baseline.

Prior to the initiation of QR administration, baseline blood pressure was not significantly different among all control and QR-treated rats. To examine the effect of QR on blood pressure in SHR rats, we measured the SBP, DBP, and MAP of all groups once weekly for seven weeks. As expected, control rats showed a progressive rise in blood pressure that is characteristic of the developmental phase of hypertension in SHR (Figure 3A). Compared to a change of +22% in SBP in the control group, low, medium, and high QR doses resulted in a change of +23%, −3.0%, and −10.5%, respectively (Figure 3B). On the other hand, while DBP increased by +33% in the control group, low, medium, and high QR doses resulted in a change of +24%, −8.6%, and −13.5%, respectively (Figure 3B). Similarly, compared to a change of +10% in MAP of the control group, low, medium, and high QR doses resulted in a change of +28%, +0.80%, and −13.5%, respectively (Figure 3B).

Although low-dose QR did not result in significant changes in blood pressure, administration of medium- and high-dose QR resisted the rise in blood pressure observed in the untreated SHR. Only the high-dose QR resulted in a significant decrease in blood pressure from week 1 and through week 7 of treatment (*p* < 0.05) compared to their baseline values and the control group. A similar significant reduction in blood pressure was detected in the medium QR dose group only after five weeks of treatment. Comparing blood pressure values by the end of the seven-week treatment to the baseline record, control rats demonstrated a significantly higher blood pressure which is expected in this animal model (Figure 3B). Low-dose QR was unable to affect this trend of progressive rise in blood pressure, and therefore rats in this group had a significantly higher blood pressure compared to the baseline values. Medium-dose QR, on the other hand, resulted in slowing down the observed increase in blood pressure but was not effective enough to cause a significant reduction in blood pressure. Therefore, rats in this group had blood pressure values not significantly different from the baseline readings (Figure 3B). Rat treatment with high-dose QR for seven weeks not only resulted in resisting the rise in blood pressure, but also caused a significant reduction in blood pressure compared to the baseline values.

### 2.2. Effect of QR Treatment on 20-HETE Formation in Renal Microsomes

**Figure 4:**
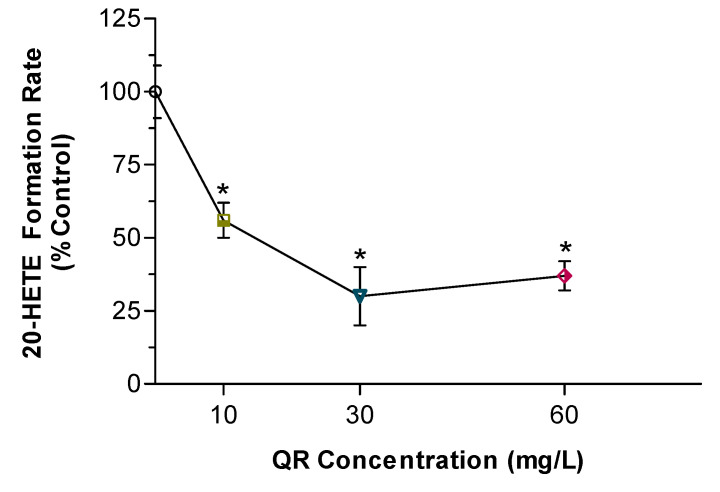
Effect of QR on the rate of 20-HETE formation in rat kidney microsomes. Mean 20-HETE concentration from rat kidney microsomes (*n* = 8/group) treated for 7 weeks with QR in drinking water at different concentrations, expressed as the percentage of the control group. All groups were compared using one-way ANOVA followed by multiple comparisons. * Significant difference from control with *p* < 0.05.

Previous studies have demonstrated the implication of cortical CYP metabolites of arachidonic acid in the regulation of blood pressure [3]. Effective inhibition of the CYP4A-mediated formation of the potent vasoconstrictor metabolite 20-HETE was found to decrease blood pressure in SHR in a dose-dependent manner [8]. To examine the potential inhibitory effect of sub-chronic oral administration of QR on the 20-HETE formation rate, as a marker for CYP4A activity, 20-HETE was measured in rat renal microsomes after seven weeks of exposure to QR in the drinking water at low, medium, and high concentrations using an LC-MS/MS method as described in the Methods section. Figure 4 illustrates a trend of dose-dependent inhibition of 20-HETE formation. All treatment doses of QR resulted in significant inhibition of the 20-HETE formation rate in renal cortical microsomes. The formation of 20-HETE was significantly inhibited to 60%, 30%, and 35% of the control value by the low, medium and high doses of QR, respectively (Figure 4).

### 2.3. Effect of QR Treatment on Soluble Epoxide Hydrolase (sEH) Activity

**Figure 5:**
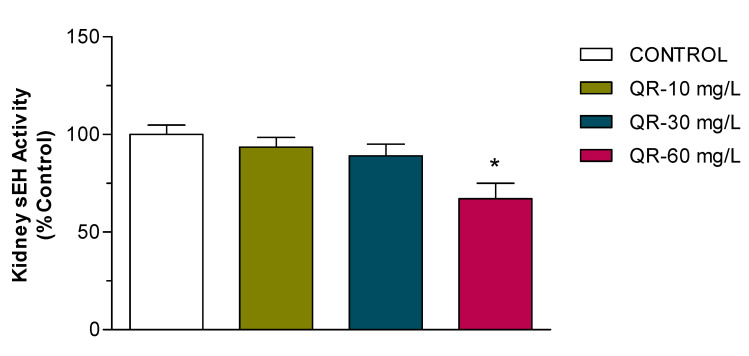
Effect of QR on the activity of kidney soluble epoxide hydrolase (sEH). Mean sEH activity from rat kidney cytosols (*n* = 8/group) treated for 7 weeks with QR in drinking water at different concentrations, expressed as the percentage of the control group. All groups were compared using one-way ANOVA followed by multiple comparisons. * Significant difference from control with *p <* 0.05.

Despite the reported cardioprotective effect of eicosanoids, they undergo rapid hydrolysis to inactive metabolites by the enzyme sEH. Therefore, we investigated the potential inhibitory effect of QR on the activity of this enzyme in kidney cytosolic fractions from control and QR-treated SHR. The results shown in Figure 5 indicate that administration of the high dose of QR results in 35% inhibition of sEH activity (*p* < 0.05). Administration of the low and medium doses, on the other hand, did not result in significant inhibition of sEH activity.

**Figure 6:**
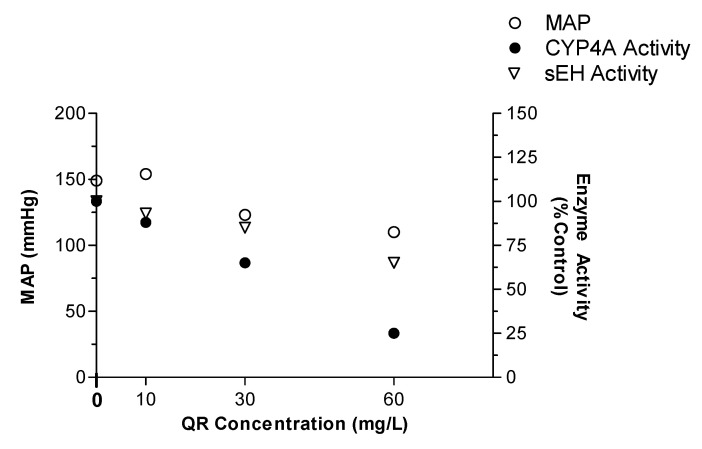
The association between dose-dependent changes in CYP4A and sEH activity, and the changes in mean arterial blood pressure (MAP). Treatment rats received QR in their drinking water at concentrations of 10, 30, and 60 mg/L, for 7 weeks. Control rats received drinking water only (i.e., QR concentration is 0 mg/L).

To examine the impact of changing the activity of CYP4A and sEH on the overall changes in blood pressure caused by different QR doses, we determined the correlation between these variables. As illustrated in Figure 6, the changes in MAP were considerably correlated (Pearson r; −0.65 and *p*; 0.034) with the reduction in CYP4A activity, as indicated by the decrease in the 20-HETE formation rate. Namely, while the medium and high QR doses reduced MAP by approximately 19 and 31%, CYP4A activity was reduced by 35 and 75%, respectively. Similarly, a significant correlation (Pearson r; −0.86 and *p*; 0.014) was established between changes in MAP and changes in sEH activity in a QR dose-dependent manner. Specifically, compared to 19 and 31% reduction in MAP by the medium and high QR doses, sEH activity was reduced by 15 and 35%, respectively (Figure 6).

## 3. Discussion

This study has shown that sub-chronic administration of QR reduces blood pressure in young SHR rats, and this effect was associated with the modulation of arachidonic acid in the kidney.

QR is a naturally occurring flavonoid that exhibits a wide range of biological actions, including cardioprotective, antioxidant, and anti-inflammatory properties [20]. The majority of the published studies that have examined the beneficial effects of acute exposure to QR support its protective effect in cardiovascular diseases. However, there is little information about its underlying mechanism of action and the impact of chronic exposure to QR in a rat model of hypertension. We elected to administer QR in drinking water rather than oral gavage or subcutaneous injection to (1) avoid inducing any stress-related effects on blood pressure and (2) to simulate the commonly used route of QR administration. We introduced QR to rats in their drinking water. Due to its poor water solubility, and to avoid exposing animals to organic solvents, QR solutions were prepared using Lutrol F127 Pluronic^®^, a GRAS excipient recognized by the FDA and commonly used in commercial products for human consumption at concentrations of 5 % or below. To account for any potential effect of the solvent, the control group received a QR-free 5% Pluronic^®^ F127 solution. Our stability studies indicate that the QR solution is stable at room temperature for at least five days. Therefore, fresh QR solutions were made in 5% Pluronic^®^ F127 every three days. Further, our previous studies have indicated that the oral bioavailability of QR in a SHR rat model is comparable to reported bioavailability data in humans [21]. The selection of the QR concentration in drinking water was designed to mimic the average daily servings of quercetin, i.e., clinically relevant doses, taking into consideration the average daily water intake and predicted oral bioavailability of QR. Considering the average rat weight and average daily water intake, QR concentrations used in this study provide approximately 2, 6, and 12 mg/kg QR, respectively. These doses are equivalent to 0.25–1.5 times the average human daily intake on a weight to weight basis [22]. It should be noted that few studies have reported a similar antihypertensive effect of QR, however they utilized higher doses (10 mg/kg) [23,24] or administered QR by an intraperitoneal route instead of oral administration [25].

To the best of our knowledge, this study is the first to demonstrate that the QR-mediated antihypertensive effect is associated with the modulation of AA metabolism in the kidney. Particularly, the efficacy of the seven-week treatment with QR in reducing the expected progressive rise in blood pressure in SHR (Figure 3) was comparable to commonly used antihypertensive medications in the same rat model. For example, amlodipine (3 mg/kg for four weeks), telmisartan (3 mg/kg for eight weeks), hydrochlorothiazide (10 mg/kg for eight weeks), and lisinopril (10 mg/kg for eight months) resulted in an average MAP reduction of 15%, 17%, 18%, and 20%, respectively [26,27,28].

Major products of renal AA metabolism, namely 20-HETE and EETs, play a key role in arterial blood pressure regulation [11,13]. Several studies have suggested that elevated production of 20-HETE has a pro-hypertensive effect, through its strong vasoconstrictive effect on the renal afferent arteriole and promoting sodium retention, in the kidney of SHR, which may contribute to the rapid rise in mean blood pressure in this rat model [15]. Our study shows that sub-chronic exposure of SHR to medium- and high-dose QR results in a reduction in blood pressure, and this effect was associated with a similar effect on the activity of renal CYP4A, the enzyme responsible for the formation of 20-HETE (Figure 4). In support of our results, administration of inhibitors of CYP4A1/2 such as 1-aminobenzotriazole (ABT) [8] or CYP4A1 antisense oligonucleotide [9] also reduces blood pressure and renovascular tone in SHR.

A plethora of evidence indicates that the rapid hydrolysis of EETs, potent vasodilators, and natriuretic agents by the enzyme sEH to biologically inactive diols significantly contributes to the development of hypertension in SHR compared to their normotensive WKY control rats [17,29]. Therefore, targeted disruption of sEH and enhancing the availability of EETs have become a promising approach for the treatment of hypertension and restoring the dilation function of the endothelium. Studies by our laboratory and others demonstrate that chronic inhibition of sEH for one–six weeks lowers blood pressure and ameliorates organ damage associated with hypertension [6,10,16]. The data from our current study indicate, for the first time, that medium and high doses of QR reduce blood pressure in SHR, but only high-dose QR causes significant decreases in the renal activity of sEH (Figure 5). Studies aimed to develop sEH inhibitors as a potential treatment for cardiovascular and renal diseases were initiated in the 2000s. The first-generation inhibitors were chalcone oxides and glycidol derivatives, and were associated with severe side effects such as inhibition of glutathione and glutathione-S-transferases [30]. Recently, large molecular weight ureas, carbamates, and amide inhibitors were developed based on X-ray structures of murine and human sEH enzyme [31]. Despite their potent antihypertensive effect, therapeutic use of these inhibitors was hampered by their poor physicochemical and unfavorable pharmacokinetic properties. A recent focus on nature’s toolbox has resulted in the discovery and development of promising and clinically useful drug candidates such as capsaicin and curcumin. Unfortunately, natural products do not currently play a major role in the area of cardiovascular diseases, especially hypertension. Identification of natural products as sEH inhibitors may have potential in the development of new therapies against hypertension, and possibly other devastating conditions. Naturally occurring flavonoids such as QR have demonstrated strong antioxidant, chemo-preventive, and anti-inflammatory effects [18,23]. The data presented in this manuscript demonstrate significant sEH inhibition and antihypertensive effects of QR so that it may be deployed as a cost-effective, stand-alone, or complementary approach for the treatment of hypertension and the prevention of organ damage induced by uncontrolled high blood pressure.

Whether the observed antihypertensive effect of QR is solely attributed to its effect on AA metabolism and inhibition of both CYP4A and sEH enzymes in the kidney is not yet established (Figure 6). For example, 20-HETE has been shown to be a substrate for a wide variety of enzymes such as CYP2C, alcohol dehydrogenase, lipoxygenase, cyclooxegenase, glucuronosyltransferase, and β-oxidation pathways [3]. It should be noted that QR has shown a potent antioxidant effect in SHR, which may play a role in its antihypertensive effect [23]. However, if the antihypertensive effect of QR is due exclusively to its antioxidant properties, then similar antihypertensive properties should be observed with other antioxidants. However, several clinical trials testing the effects of antioxidants such as vitamin E and vitamin D on hypertension revealed a non-significant reduction in blood pressure [32,33,34,35]. Therefore, the observed antihypertensive effect of QR could be attributed to a combination of its antioxidant properties as well as its effect on AA metabolism.

## 4. Materials and Methods

### 4.1. Materials

Arachidonic acid, 20-HETE, and d4-20-HETE (the internal standard) were obtained from Cayman Chemical Company (Ann Arbor, MI, USA). Quercetin and all chemicals used in enzymatic assays were purchased from Sigma-Aldrich (St. Louis, MO, USA). Lutrol F127 Pluronic^®^ (F127) was kindly provided by BASF (Florham Park, NJ, USA). Organic solvents used in the LC-MS/MS analysis were HPLC-grade (Fisher Scientific, Pittsburg, PA, USA).

### 4.2. Animals

Thirty-two 5-week-old male SHR (Charles River Laboratories, Wilmington, MA, USA) were housed two per cage under constant temperature (24 ± 1 °C), 12-h dark–light cycle, and a standard laboratory chow with free access to tap water. All animals were allowed 2 weeks to adapt to the new housing environment and to ensure stable and consistent blood pressure readings before the experiments were started. Studies were carried out in accordance with the protocols and guidelines established by the Institutional Animal Care and Use Committees of Pacific University (Project IACUC R-0027, approved January 2018).

### 4.3. Preparation of Quercetin (QR) Solutions

Due to QR’s poor water solubility, QR solutions were prepared in Lutrol F127 Pluronic^®^ as a vehicle at concentrations of 10, 30, and 60 mg/L as explained previously [18]. According to our preliminary stability studies of QR in Pluronic^®^ F127, fresh QR solutions were made in 5% F127 Pluronic^®^ every 3 days.

### 4.4. Quercetin (QR) Treatment

Animals were adapted to the housing conditions then randomly divided into 4 experimental groups (*n* = 8). Group 1 served as the vehicle control group and received QR-free 5% Pluronic^®^ F127 solution. Groups 2, 3, and 4 served as treatment groups and received 5% Pluronic^®^ F127 solution containing QR at concentrations of 10, 30, and 60 mg/L, respectively.

### 4.5. Blood Pressure Measurements

Systolic (SBP), diastolic (DBP), and mean arterial (MAP) blood pressure were measured at 7-day intervals for 7 weeks in conscious, pre-warmed, restrained rats using the CODA™ tail-cuff blood pressure system. (CODA-HT4, Kent Scientific, Torrington, CT, USA) as described elsewhere [7]. At least 10 stable blood pressure determinations were made in every session and the results are reported as the average of the blood pressure values obtained from individual rats. Preliminary studies in our laboratory demonstrated a close correlation between blood pressure measurements obtained with the non-invasive tail-cuff method employed in our laboratory and values obtained by direct measurements using an indwelling catheter. Body weight was measured weekly immediately after measuring blood pressure.

### 4.6. Tissue Collection

Following the 7-week treatment and weekly measurement of blood pressure, animals were killed, and the kidneys were rapidly removed, rinsed with ice-cold saline, and snap-frozen in liquid nitrogen, then stored at −80 °C until use. Kidney microsomal and cytosolic fractions were prepared from the renal cortex as described previously [18]. Microsomal and cytosolic protein concentrations were determined in triplicate as described before using bovine serum albumin as a calibration standard.

### 4.7. Quantification of 20-HETE Metabolite in Rat Kidney Microsomes

To investigate the effect of QR on CYP4A activity, the 20-HETE formation rate was measured in renal cortical microsomes prepared from control and QR-treated rats according to Lasker et al. [2] with slight modifications. Briefly, the incubation mixtures contained microsomal protein (500 µg), arachidonic acid (100 mM), MgCl_2_ (10 mM), and KCl (150 mM) in 1 mL of 100 mM potassium phosphate buffer (pH 7.4). The reaction was initiated by adding NADPH (1 mM) and carried out for 15 min at 37 °C, then terminated with 20 µL of 2N HCL. Arachidonic acid and 20-HETE were extracted thrice with ethyl acetate, the combined organic phase was evaporated under nitrogen and the dry residue was reconstituted in 400 µL of an acetonitrile/water/formic acid (59.3:40:0.70%) mixture. Chromatographic separations were carried out by using a Kinetex C_18_ column (100 × 4.6 mm, 2.6 µm); (Torrance, CA, USA) maintained at 40 °C with 10 µL injection volume. The mobile phase was pumped using a binary gradient elution consisting of solvent (A): 0.1% formic acid in acetonitrile, and solvent (B): 0.1% formic acid in 50% water and 50% methanol (60:40, *v*/*v*) delivered at a flow rate of 0.8 mL/min to separate 20-HETE and its isotope IS (d4-20-HETE). Air (zero grade) was the nebulizer gas, whereas nitrogen was used as the curtain, auxiliary, and collision gases. The source/gas-dependent parameters were as follows: curtain gas, 30 psi; collision gas, 8 psi; ion spray voltage, 2000 V; medium temperature, 500 °C; ion source gas one, 40, and gas two, 20 psi. The following precursor-to-product ion pairs transitions at *m*/*z* 318.91 →275.18 and *m*/*z* 324.9 →281.3 for 20-HETE and, *4*-20-HETE (IS), respectively, are represented in Table 1. 20-HETE formation, expressed as nmol/min/mg protein, was calculated from standard curves run on the same day using authentic 20-HETE samples.

### 4.8. Measurement of Soluble Epoxide Hydrolase Activity using Fluorescence Assay

To examine the effect of QR on the sEH activity in kidney cytosol, Epoxy Fluor 7 (Cayman Chemical Co., Ann Arbor, MI, USA) was utilized as a sensitive fluorescent substrate. Formation of the highly fluorescent metabolite was monitored at excitation and emission wavelengths of 330 and 465 nm, respectively, on a Synergy2^®^ microplate reader using Gen5 Software (BioTek, Winooski, VT, USA) as described previously [36].

### 4.9. Data Analysis

Measurements of blood pressure and enzyme activity are presented as mean ± standard error of the mean (SEM). Statistical differences in enzyme (CYP4A and sEH) activity and in blood pressure between groups were evaluated by one-way analysis of variance (ANOVA), followed by Tukey’s post hoc test to detect differences between groups. A probability of *p* < 0.05 was considered statistically significant. Statistical analysis and graphical representation were conducted using GraphPad Prism (version 5.0, San Diego, CA, USA).

## 5. Conclusions

In summary, our results indicate that sub-chronic administration of medium-dose QR slowed down the progressive rise in blood pressure that is normally observed in SHR during development. Administration of the high QR dose, on the other hand, resulted in a significant reduction in blood pressure compared to control untreated rats. Additionally, we demonstrated, for the first time, that QR administration affected the metabolism of AA in the kidney by inhibiting the activity of renal CYP4A as indicated by decreasing the rate of 20-HETE formation and inhibiting the enzyme sEH. Collectively, these findings indicate that the antihypertensive effect, and thus cardioprotective properties, of QR are mediated, at least partially, by its effect on AA metabolism.

## Figures and Tables

**Table 1:** LC-MS/MS parameters selected for the quantification of 20 HETE and d4-20 HETE (IS).

Analyte	Q1^a^ (m/z)	Q3^b^ (m/z)	DP^c^ (V)	EP^d^ (V)	CE^e^ (V)	CXP^f^ (V)
**20- HETE**	318.91	275.18	−60	−10	−22	−11
**d4−20-HETE (IS)**	324.90	281.30	−80	−10	−22	−11

^a^ Q1, precursor ion; ^b^ Q3, product ion; ^c^ DP, declustering potential; ^d^ EP, entrance potential; ^e^ CE, collision energy; ^f^ CXP, cell exit potential.
